# Evaluation of polycaprolactone as a new sorbent coating for determination of polar organic compounds in water samples using membrane–SPME

**DOI:** 10.1007/s00216-014-8328-0

**Published:** 2014-11-22

**Authors:** Łukasz Marcinkowski, Adam Kloskowski, Agata Spietelun, Jacek Namieśnik

**Affiliations:** 1 Chemical Faculty, Department of Physical Chemistry, Gdansk University of Technology, Narutowicz St., Gdańsk, 80-233 Poland; 2 Chemical Faculty, Department of Analytical Chemistry, Gdansk University of Technology, Narutowicz St., Gdańsk, 80-233 Poland

**Keywords:** Sample preparation, Polycaprolactone, Sorption, Solid phase microextraction, Triazines, Water samples

## Abstract

Commercially available solid-phase microextraction fibers used for isolation of polar analytes are based on the adsorption phenomenon. In consequence, typical limitations bonded with analytes displacement and matrix effects are very frequent. In the present study, alternative solution is described. Polycaprolactone (PCL) was used for the first time as sorbent to isolate polar organic compounds from water samples using the membrane–solid-phase microextraction (M-SPME) technique. In this technique, due to protective role of the mechanically and thermally stable polydimethylsiloxane (PDMS) membrane, internal polar coating might be melted during extraction and desorption of analytes. In consequence sorbents with low melting points like a PCL might be utilized. Based on chromatographic retention data, triazines were selected as a model compounds for evaluation of the sorptive properties of the polycaprolactone. Applying the screening plan and central composite design, statistically significant parameters influencing extraction efficiency were determined and optimized. The analysis of variance confirmed the significant influence of temperature, salt content, and pH of samples on the extraction efficiency. Besides the new PCL/PDMS fiber, a commercial fiber coated with divinylbenzene/polydimethylsiloxane (DVB/PDMS) was used for comparative studies. The results obtained showed that PCL is an interesting sorbent which can be successfully applied for isolation of polar organics from aqueous matrices at a broad range of analytes concentration. The determined detection limits of procedure based on the novel fiber enable its application at the concentration levels of triazines recommended by the US EPA standards. The practical applicability of the developed fiber has been confirmed by the results based on the analysis of real samples.

## Introduction

Determination of polar organic compounds in water samples is still a challenge for the analysts. Efficient isolation of polar compounds requires utilization of the polar sorbents. However, when adsorbents are applied, competitive adsorption of water may be a serious problem in quantification of the results of analysis. From this perspective, application of the liquid or liquid-like materials as the sorbent phase seemed to be a way to bypass this problem. Unfortunately, many useful materials cannot be applied as extraction phases due to their mutual solubility in water. Such possibility was opened with development of the membrane–solid-phase microextraction technique, where polar phase is physically separated from aqueous phase with a nonpolar membrane. One group of polar analytes important from environmental point of view are triazines—compounds still being used in herbicidal preparations. The problem associated with the use of triazine herbicides for protecting cultivated plants against weeds is a necessity of control of herbicide residues in the environment. Triazine compounds and their derivatives are characterized by their stability in the environment, particularly in soil, ability to be accumulated in plants, and high toxicity [1]. One of the primary activities permitting reduction of the negative impact of triazines on human health is the continuous monitoring of their presence in environmental samples, particularly in the samples of drinking water and food. According to the existing regulations (98/83/CE Directive), the maximal allowable content of herbicides (including triazines) in drinking water is 0.1 μg L^−1^ [2].

The SPME technique, which is characterized by high simplicity, has been widely used in analytical practice to sample a broad spectrum of analytes from media of complex composition [3]. However, the extraction efficiency of this technique, due to the small amount of sorptive phase, strongly depends on the type of the sorption material. Commercially available polyacrylate (PA) [4–7], Carbowax/divinylbenzene (CW/DVB) [8], divinylbenzene/polydimethylsiloxane (DVB/PDMS) [9–12], and PDMS [13, 14] fibers are applied to extract triazines from aqueous samples. These fibers have some limitations, e.g., low affinity of PDMS coating towards polar analytes. In the case of other fibers, the main problem is related to the adsorption mechanism involved. In this case, there is no simple relationship between the amounts of retained analytes and their concentrations in the sample what makes quantitative analysis more difficult compared to sample preparation based on partitioning mechanism. What is also important is that the application of adsorbents creates a risk of displacement effects and makes extraction process more sensitive to changes in matrix composition [15].

Therefore, the major trend in the development of SPME technique is the elaboration of novel stationary phases in order to achieve the highest possible extraction efficiency of polar analytes. To this end, the materials dedicated to specific compound types are applied to extract triazines by the SPME technique, i.e., molecularly imprinted polymers (MIP) [16, 17], sol–gel synthesized sorbents [18], and dodecylsulfate-doped polypyrrole (Ppy-DS) synthesized by using electrochemical techniques [19].

In this paper, we present results of examination of the polycaprolactone (PCL) as an extractant for isolation and preconcentration of the polar organic compounds from water samples. PCL, due to its low melting point, has never been applied for this purpose. To solve this problem, the system based on the separation of the extraction phase from the sample with hydrophobic polymer was developed [20, 21]. Application of the polydimethylsiloxane as a membrane enables dissolution of the extraction material in sample matrix and maintains the shape of the coating. In this way, it is possible to apply, as the polar retaining medium, materials which, so far, have been excluded from these applications due to their solubility in water and/or rheological properties. External hydrophobic coating acts as a membrane, which makes our system similar to the well-known membrane separation techniques. The preliminary study, based on retention data of McReynolds compounds, showed that triazines may be a type of compound with potentially high affinity for PCL. Thus, in the present study, seven triazines were utilized as a model analytes. A commercially available SPME fiber coated with DVB/PDMS was used for comparative purposes. In both systems, the optimization of extraction conditions was performed by using chemometric techniques and the linear range, limits of detection, and matrix effects were determined.

## Materials and methods

### Reagents and analytical standards

HPLC-grade methanol (Sigma-Aldrich, Germany) was used to prepare polycaprolactone and triazine stock solutions. Chloroform (POCH, Poland) was used to prepare squalane solution. The 100 μg mL^−1^ standard solutions of triazine compounds in methanol were purchased from Sigma-Aldrich (Germany). The McReynolds compounds (Sigma-Aldrich. Germany), i.e., benzene, 1-butanol, 2-pentanone, nitropropane, and pyridine were of analytical grade, NaCl and the components of buffer solutions were obtained from POCH (Poland). Squalane, used as the stationary phase in packed GC column, was purchased from POCH (Poland). Polycaprolactone of 99.9 % purity and molecular weight of 14,000 g mol^−1^, used to produce the M-SPME fiber and as the stationary phase in a chromatographic column, was purchased from Carbomer Inc. (USA). Polydimethylsiloxane, used as the nonpolar membrane in M-SPME fibers, was obtained from Dow Corning (USA) in the form of a two-component prepolymer kit Sylgard® 184. Divinylbenzene/PDMS fibers with 65-μm-thick coating were purchased from Supelco (Bellefonte, USA). Optical fibers of 150 μm glass core diameter were purchased from Cezar Int (Poznań, Poland). The polyamide protective layer was removed from these fibers using concentrated sulfuric acid. High-purity nitrogen (99.999 %), purchased from Linde Gas (Poland), was used as the carrier gas. Chromosorb W HP-DMCS (80/100 mesh, Sigma-Aldrich, Germany) was used as the solid support of stationary phase in a capillary column. Deionized water was obtained from the Milli-Q water purification system (Millipore®, USA).

### Column packing

In this study, two packed columns were used. One column was filled with solid support coated with PCL (the latter used also as the polar phase in M-SPME), while the second column with solid support and squalane as nonpolar stationary phase. Utilized coating procedure is typical for preparation of the packed GC column ensuring homogenous coverage of the support material [22]. Both columns were prepared according to the similar procedure although different solvents were used to make the solutions of polycaprolactone and squalane (see previous section).

In order to coat the support, solutions of polycaprolactone and squalane were prepared. Next, ca. 4 g of Chromosorb was introduced to the PCL or squalane solutions. Subsequently, solvents were removed using a rotary evaporator. In the case of PCL, the evaporation was conducted for 12 h at 50 °C under low vacuum. For squalane, the evaporation took place at 40 °C and under atmospheric pressure. The coverage of the support was evaluated gravimetrically after the evaporation procedure; it reached 36.4 and 39.6 % for polycaprolactone and squalane, respectively. Such high coverage prevents residual interactions between the investigated analytes and support. The packed columns were made from a 1 m long (2.1 mm I.D.) stainless steel tube. After packing, both ends of the column were plugged with glass wool. The mass of stationary phase was determined gravimetrically. Prior to analytical use, the columns were preconditioned in the GC oven until a stable baseline has been obtained (ca. 12 h at 130 °C with an inert gas flow at 5 mL min^−1^).

### Preparation of the M-SPME fibers

**Fig. 1:**
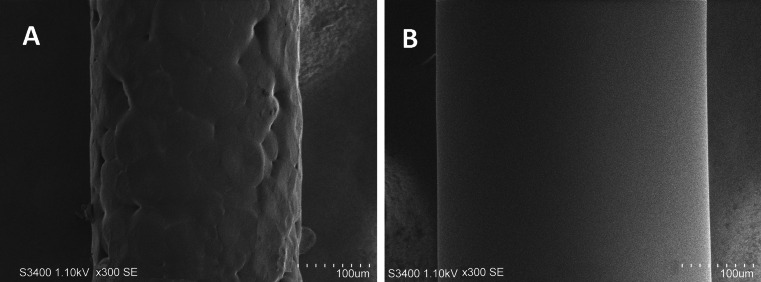
Scanning electron microscopy images of PCL (**A**) and PCL/PDMS (**B**) coatings

Two-phase fibers were made by the dip-coating technique. In the first step, the glass fiber was immersed in a molten polymer to the depth of 12 mm and then withdrawn at a precisely controlled rate using homemade setup (precision of 1 % of the linear velocity). As a result, the fiber surface was coated with the polymer film which thickness depended on the polymer temperature and rate of withdrawing the fiber. The PDMS coating was obtained in the same way, although the procedure had to be repeated a number of times. The coating procedure has been described in detail in previous paper [20]. In the case of polycaprolactone, the polymer was kept at 67 ± 0.01 °C, which is slightly above its melting point of 65 °C. The thickness of both polar and nonpolar layers was measured by using an optical microscope equipped with a digital camera (total system resolution ±1 μm). The layer on fiber used in this study had an average thickness of 67 ± 3 μm for PCL and a 37 ± 5 μm thick for PDMS coating. In Fig. 1, images of PCL and PCL/PDMS coatings and obtained using scanning electron microscope (Hitachi, model S-3400 N) are shown. Considering the length of both coating layers (10.2 mm for PCL and 11.0 for PDMS) and the glass fiber diameter (150 μm), the volumes of polar and nonpolar phases were determined to be 0.47 ± 0.02 and 0.41 ± 0.04 μL, respectively. A fiber coated with polydimethylsiloxane only, with the coating layer volume of 0.40 ± 0.035 μL was also used (volume close to the volume of PDMS in PCL/PDMS fiber). This fiber was made from the same prepolymer as the M-SPME fiber. The PCL/PDMS fiber’s lifetime was determined by monitoring the change of extraction efficiencies obtained by performing repeated extractions under definite experimental conditions with a single fiber. It was established that a single fiber could be used with no obvious decline of performance for about 60 extraction/desorption cycles.

### Extraction procedure

The standard solutions were prepared based on previously made aqueous solutions of NaCl with known concentrations and stable pH. The solutions were prepared in vials closed with hole caps with PTFE/silicone septa. The phosphate (pH ≥ 7) and citrate (pH < 7) buffers were used to adjust pH of the solutions. The volumes of the standard solutions were 15 mL. Stock solutions of mixture of the analytes were prepared in methanol. The concentrations of the stock solutions were chosen in such a way as to ensure that their volumes added to the matrix could be neglected (max 50 μL). Prior to addition of a stock solution, the matrix was kept at a stable temperature for 15 min in a homemade thermostat with a precision of ±0.2 °C and after addition, thermostating was continued for another 15 min and, subsequently, the fiber was immersed in so prepared standard solution. During the incubation in a thermostat and extraction, samples were stirred with a glass covered, magnetic stirring bar at a maximum speed of 1200 rpm. The commercial SPME fibers were preconditioned in accordance with the manufacturer’s recommendations. All the M-SPME fibers were preconditioned before the first use by heating in the GC injector at 210 °C for 1 h.

### GC analysis

The experiments were conducted using Agilent 7890A gas chromatograph equipped with an FID detector (Palo Alto. USA). Desorption of analytes took place inside the GC injector at 200 °C for the PCL/PDMS fibers, and at 230 °C for the commercial fibers. The different desorption temperatures resulted from the manufacturers’ recommendations regarding the maximum allowable temperature for the commercial fiber and the PDMS membrane of M-SPME fiber. The detector temperature was set at 250 °C. The GC analytical column was 5Sil (30 m × 0.32 mm I.D., 0.25 μm film thickness). Temperature program: 60 °C for 6 min; raised to 150 °C at 10 °C min^−1^; raised to 175 °C at 1 °C min^−1^; raised to 220 °C at 30 °C min^−1^ and was held for 3 min. Nitrogen was used as the carrier gas at a constant flow rate of 1 mL min^−1^.

## Results and discussion

### Preliminary investigations

**Fig. 2:**
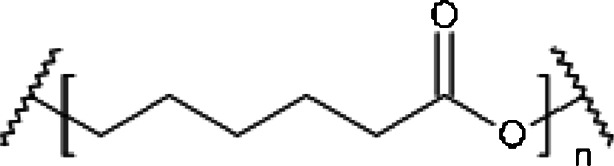
Chemical structure of polycaprolactone

In Fig. 2, the structural formula of polycaprolactone is shown. The presence of a strongly polar ester group is a decisive factor with regard to polycaprolactone’s potential for being an extract of polar compounds.

At the same time, PCL is not considered to be highly polar for example in comparison to polyethylene glycol which had been previously used in the M-SPME technique. The practical application of this polymer as a polar sorbent in the M-SPME device requires, however, that the two additional conditions are fulfilled. First, the polymer has to be characterized by a properly low melting point (far below the boiling point of water), which enables the isolation of analytes based on partitioning mechanism. Secondly, the polymer should display suitable thermal stability which would allow for the release of analytes via thermal desorption. In this study, polycaprolactone with the mean molecular weight of 14,000 g mol^−1^ was used. Because the melting point does not exclusively depend on the mean molecular weight, but also on its distribution in the given polymer batch, additional measurements were performed. PCL melting point was determined by scanning calorimetry, using the DSC Phoenix 204 calorimeter (Netzsch, Germany) in an inert gas atmosphere. The value found was ca. 65 °C (peak value). Moreover, no signs of thermal decomposition were detected in the investigated polymer within the temperature range used during the measurements, i.e., up to 250 °C.

The chemical formula of polycaprolactone points to its polar character. However the polymer’s chemical formula is not a parameter which would precisely define the group of analytes for which PCL could be the most suitable medium. Therefore, a study of molecular interactions characteristic for polycaprolactone was conducted. For this purpose, the methodology based on McReynolds constants was applied. This methodology is often used in chromatographic techniques for determining the polarity of stationary phases. In general, the procedure is based on determining the retention times of compounds that represent different types of molecular interactions. A column containing solid support coated with the investigated compound, and squalane as a nonpolar phase is used. In this way, the compound-specific molecular interactions of the stationary phase and its polarity can be determined. The model compounds used in this study and their typical interactions are listed in Table 1.

**Table 1:** Molecular interactions of the tested McReynolds compounds

Compound	Type of interactions
Benzene	Weak dispersion forces and polarizability of the phase. π-π interactions
1-Butanol	Hydrogen-bonding ability of the phase
2-Pentanone	Polarizability and partial dipolar character of the stationary phase
Nitropropane	Electron donor, electron acceptor, and dipolar character of the phase
Pyridine	The acidic character of the phase

Determination of McReynolds constants requires that Kovats indices (Eq. 1) are calculated based on the adjusted retention times (correction for hold-up retention time) of a number of *n*-alkanes and model compounds.

1 $$ I=100Z+\frac{100\left[ \log {t}_{\mathrm{R}}^{\prime }(i)- \log {t}_{\mathrm{R}}^{\prime }(z)\right]}{ \log {t}_{\mathrm{R}}^{\prime}\left(z+1\right)- \log {t}_{\mathrm{R}}^{\prime }(z)} $$

where: *Z* is the number of carbon atoms in *n*-alkane with the adjusted retention time *t*_R_′(*z*); *t*_R_′(*i*) is the adjusted retention time of tested compound; *t*_R_′(*z*) is the adjusted retention time of *n*-alkane eluted before the tested compound; and *t*_R_′(*z* + 1) is the adjusted retention time of *n*-alkane eluted after the tested compound.

Kovats indices were determined for the column containing PCL-coated solid support (IPCL) and for the column with support coated with squalane as a nonpolar phase (IS). Nitrogen was used as a carrier gas, with a volumetric flow rate of 2 mL min^−1^. The hold-up time was determined by using methane as a compound not retained in the column. Column temperature was 120 °C. Finally, the values of Δ*I* for specific model compounds were calculated based on Eq. 2.

2 $$ \varDelta I= aX+bY+cZ+dU+eS $$

where weight coefficients *a*, *b*, *c*, *d*, and *e* characterize the contribution by a given test compound and *X*, *Y*, *Z*, *U*, and *S* are partial Δ*I*s for these test compounds (all characterizing the stationary phase relative to squalane). The weight coefficients for the five investigated compounds were chosen as recommended [23]: benzene (*a* = 1); 1-butanol (*b* = 1); 2-pentanone (*c* = 1); nitropropane (*d* = 1); and pyridine (*e* = 1). In this way, a set of McReynolds constants was calculated by subtracting the values of Kovats index obtained on both columns for the test compounds (see Table 2).

**Table 2:** The values of Kovats index and McReynolds constant obtained at 120 °C

Compound	*I*_S_	*I*_PCL_	Δ*I*
Benzene	677	870	193 (*X*)
1-Butanol	654	926	272 (*Y*)
2-Pentanone	662	890	228 (*Z*)
Nitropropane	676	1046	371 (*U*)
Pyridine	698	1026	328 (*S*)

Conventionally, in the case of McReynolds constants, the mean values of Δ*I* below 100 indicate nonpolar compounds, the values between 100 and 400 are characteristic for medium- polar compounds, and the values higher than 400, for strongly polar substances. In the case of PCL, the mean Δ*I* was 278, which indicates that the polymer is medium polar; with main contributions to the total polarity coming from electron donor–electron acceptor interactions. A relatively high value of Δ*I* for butanol points to some tendency to form hydrogen bonds.

Based on the obtained results, triazines have been chosen as the model analytes. The investigated triazines and their basic physicochemical parameters are listed in Table 3.

**Table 3:**
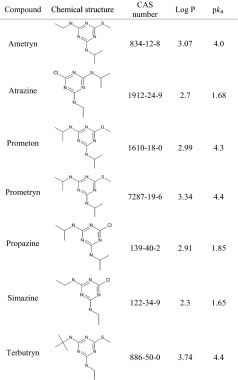
List of triazines used in the study with their chemical structures and some physicochemical properties [27]

Based on the values of logarithms of octanol–water partition coefficients (log*P*) the studied compounds can be classified as medium polar (log*P* > 3) and polar (log*P* < 3). Moreover, their dissociation constants (p*K*_a_) indicate that these compounds are weakly acidic.

### Optimization of extraction procedure

In the first step of optimization, a screening analysis was conducted, with the aim to determine the parameters which significantly influence the extraction process. The reduction of the number of parameters has a critical impact on the number of experiments which will have to be performed during optimization, particularly in full factorial design procedures. Considering available literature and the aforementioned physicochemical properties of triazines, we decided to investigate the following input variables [24, 25]:

- Extraction temperature which has a significant influence on the values of partition coefficients
- Salt content because of the possible occurrence of salt effect
- Sample pH because of the acidic properties of triazines.

In literature, the kinetic factors, such as extraction time, type of sample agitation, stirring speed, etc., are often included in the optimization process [26]. However, to avoid problems with heating of the sample, which is the often case of ultrasound agitation, we decided to use magnetic stirring for sample mixing. In consequence, the highest available rotational speed (1700 rpm) was applied to accelerate transport of the analytes towards surface of the fiber. All listed parameters influence thermodynamics in the fiber-sample system. We assume that 30 min extraction time applied during optimization experiments, although too short to reach equilibrium, allowed to draw conclusion free from kinetic aspects of extraction process. Moreover, the character of the relationship between the amount of extracted analytes (optimized dependent variable) and time is well known, and has been described by a logarithmic-like function reaching a plateau that corresponds to equilibrium. Consequently, the optimization of extraction time after establishing optimal thermodynamic conditions (which guarantee favorable partitioning of analytes in a given system) seems to be the most justified procedure, if both extraction efficiency and extraction time are considered.

#### Desorption time

Desorption time was also excluded from investigations. In our opinion, it is difficult to optimize extraction when the transfer of analytes from the fiber to chromatographic column is not quantitative. In such a situation, it is not possible to properly interpret the influence of other parameters, which are being optimized, on the efficiency of extraction. Therefore, desorption time was determined by conducting independent experiments prior to optimization. Thermal desorption of analytes from the PCL/PDMS and DVB/PDMS fibers was performed at 200 and 230 °C, respectively. Desorption efficiency was determined for a given desorption time for the analytes retained during a 30 min extraction of standard solutions (15 mL; concentration of 125 μg L^−1^ of each analyte) at 40 °C. A fivefold higher concentration of analytes used in desorption experiments, as compared to that used in extraction optimization, allows for the assumption that desorption of analytes in the latter case was also quantitative.

The verification of the quantity of released analytes was performed via chromatographic analysis of analytes that had been released from the same fiber during the subsequent desorption (desorption time 15 min). Based on the obtained results, it was established that a carry-over effect ceased in both fibers after 6 min.

Both screening and the optimization procedures were conducted under the assumption that the parameter being optimized (dependent variable) is the sum of chromatographic peak areas for all analytes.

#### Screening analysis

Screening was conducted based on the type 2^(3–1)^ elimination plan at the resolution level III. The plan was generated by using Statistica 10 software package (StatSoft, USA), with the aim to minimize the number of experiments and to determine the main effects of the investigated factors.

The design matrix was generated by random sampling; it is presented in Table 4 together with the values of variables and the results of determinations (sum of the chromatographic peak areas). In the present study, the following low (−1) and high (+1) parameter values were used: 25 and 40 °C; 0 and 20 % salt concentration; and pH 5 and 9. The commercial DVB/PDMS fiber was used.

**Table 4:** Matrix of the screening plan 2^(3–1)^, and the responses obtained for extraction of triazines from water samples determined by using the DVB/PDMS fiber (extraction time 30 min; concentration of triazines 25 μg L^−1^)

Run	Temperature [°C]	NaCl [%]	pH	Response [a.u.]
4	40	20	9	574
2	40	0	5	537
5	25	0	9	414
1	25	0	9	395
3	25	20	5	511
6	40	0	5	575
8	40	20	9	585
7	25	20	5	491

**Fig. 3:**
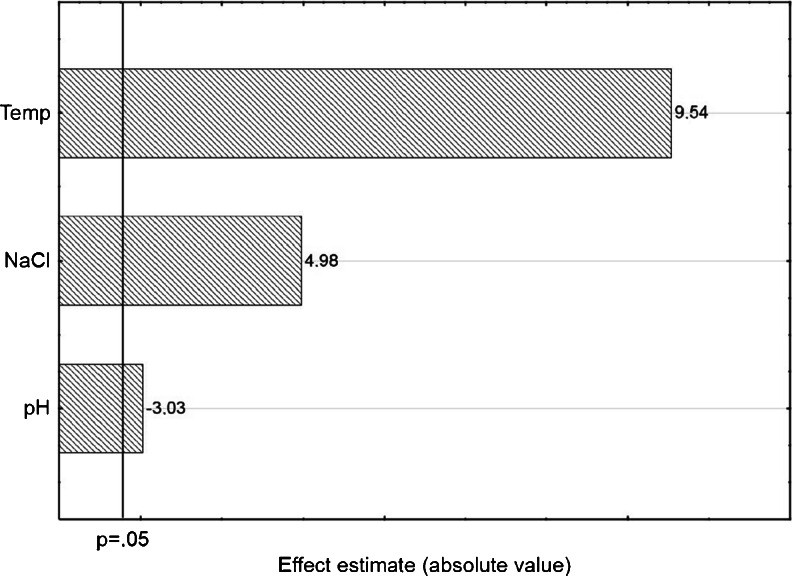
Standardized main effect Pareto chart for the 2^(3–1)^ screening plan

The statistical significance of variables was evaluated by using the analysis of variance (ANOVA); the results are presented as Pareto chart in Fig. 3. For the assumed 95 % confidence level (*p* = 0.05) depicted in the diagram, all parameters have statistically significant influence on extraction efficiency. Based on the standardized values of main effects, it can be stated that the amount of retained analytes (sum of peak areas) most strongly depends on temperature and the added salt; the influence of temperature is more significant. Both temperature and salt addition have positive effects which indicate that the extraction efficiency increases with increasing values of these parameters.

The influence of sample pH is opposite (negative effect), i.e., the extraction efficiency increases with decreasing pH. The effect of pH exceeds only slightly the assumed cut-off limit of statistical significance. To summarize, the obtained results do not warrant rejection of any parameters that have been considered during optimization.

#### Central composite design

Based on the obtained results, it was decided to adjust, in the next step of optimization, the range of temperature and salt concentration in order to cover values larger than applied previously. The new temperature range was 40–70 °C, while the new salt concentration range, 12–28 % (factorial points). The pH range had not been changed. A central composite design (CCD) was used to optimize the parameters. Moreover, the star points were added to make the design rotatable. The final plan consisted of 16 runs, i.e., 8 factorial points, 6 star points, and 2 central points. The plan was generated by random sampling. The values of independent variables corresponding to specific experiments are shown in Table 5. The table also contains the values of the response variable for the extraction procedure with the use of PCL/PDMS and commercial fibers. The presented values are means calculated from two independent measurements. In all the experiments, the extraction time was 30 min.

**Table 5:** The measured values of optimized variables and response variables (sum of peak areas) obtained as a result of triazines extraction by using the PCL/PDMS and commercial fibers (extraction time 30 min; concentration of triazines 25 μg L^−1^)

Run	Temperature [°C]	NaCl [%_*w*/*w*_]	pH	PCL/PDMS	DVB/PDMS
8	70.0	28.0	9.0	798	618
12	55.0	33.5	7.0	558	742
13	55.0	20.0	3.6	332	502
2	40.0	12.0	9.0	321	549
1	40.0	12.0	5.0	279	463
14	55.0	20.0	10.4	366	691
15 (C)	55.0	20.0	7.0	342	651
4	40.0	28.0	9.0	302	597
16 (C)	55.0	20.0	7.0	359	620
6	70.0	12.0	9.0	507	424
9	29.8	20.0	7.0	261	488
7	70.0	28.0	5.0	718	633
11	55.0	6.5	7.0	341	402
5	70.0	12.0	5.0	529	433
3	40.0	28.0	5.0	313	629
10	80.2	20.0	7.0	664	444

**Fig. 4:**
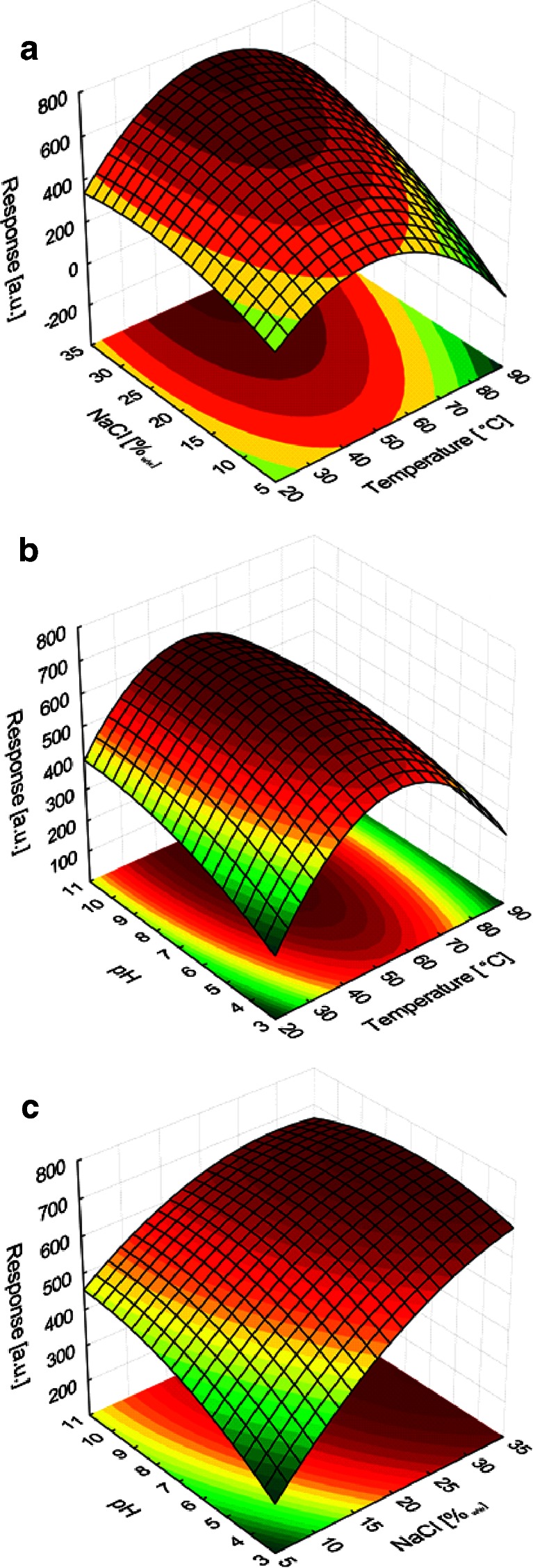
Response surfaces as functions of optimal parameters, calculated for the DVB/PDMS fiber for **a** temperature vs. salt concentration, **b** temperature vs pH, and **c** salt concentration vs pH

**Fig. 5:**
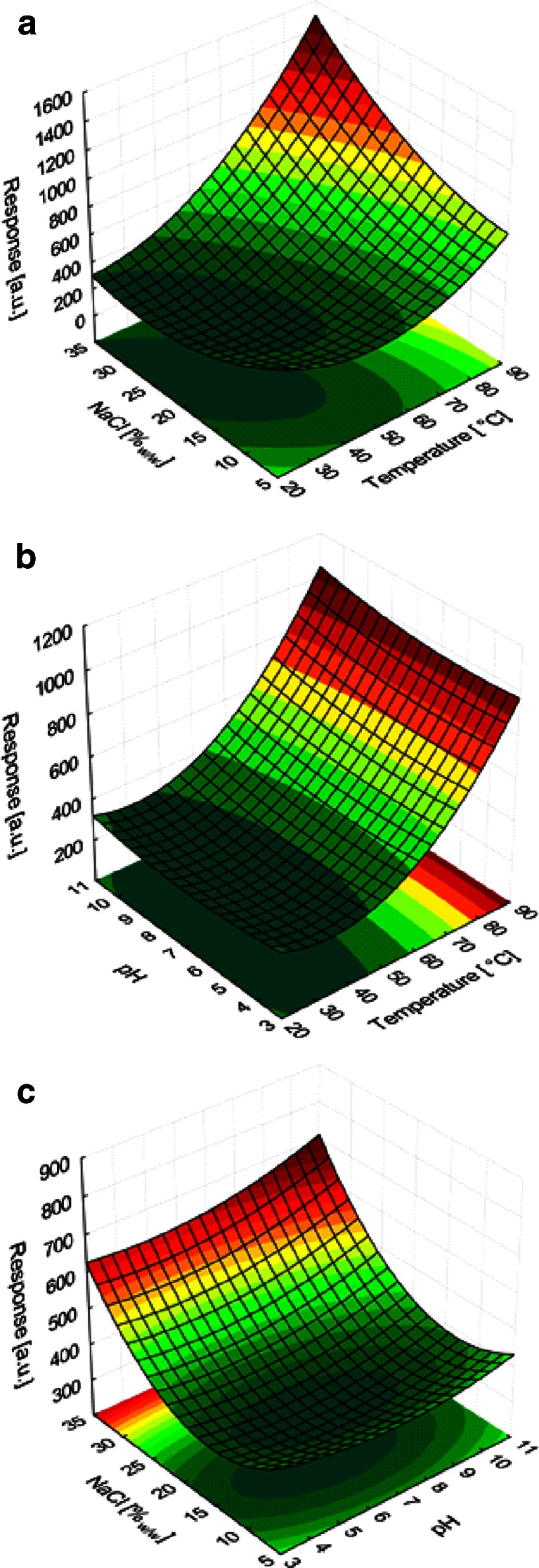
Response surfaces as functions of optimal parameters, calculated for the PCL/PDMS fiber for **a** temperature vs. salt concentration, **b** temperature vs pH, and **c** salt concentration vs pH

Response surface functions were fitted to the obtained data using the model including linear main effects, quadratic terms, and two-factor interactions. The response surfaces obtained for the commercial and PCL/PDMS fibers are presented in Figs. 4 and 5, respectively. In both cases, the response surfaces were plotted for the three combinations of independent variables; each response surface was calculated by assuming the mid-range value for the third variable. For example, pH 7 was used to calculate the response surface for the relationship between temperature and salt concentration. The models obtained for the commercial and PCL/PDMS fibers showed quite a good fit as described by the respective *R* ^2^ values of 0.921 and 0.957.

It is noticeable that there is a significant difference in response surfaces of the two fibers. In the case of commercial fiber, the optimum response for the relationship between the peak area and temperature is visibly present. Such local extremum cannot be located in the case of PCL/PDMS fiber. It is also noticeable that the addition of salt increases the extraction efficiency of both fibers; however, the influence of this parameter is not very high for the investigated range of salt concentration. Based on the results obtained for both fibers, it was assumed that the optimum salt concentration is 30 %, while the optimum pH is 7. The aforementioned salt concentration was chosen for practical reasons. At 25 °C, NaCl solution in water becomes saturated at the concentration level of ca. 35 %. The use of saturated NaCl solution creates a number of practical problems, while lowering the salt concentration to 30 % (which is in agreement with the obtained results), does not produce any drastic drop in extraction efficiency. In the case of commercial fiber, in further investigations, the value of 60 °C (a saddle point) was assumed to be the optimum. For the PCL/PDMS fiber, the optimum temperature was found to be 70 °C. Most likely, an increase in the amount of analytes retained at the higher temperatures resulted from the solid–liquid transition of polycaprolactone (melting point ca. 65 °C). However, analyzing response variables obtained for the middle line of the design (runs 9, 10, 15, 16), one might observe an opposite effect. At a temperature of 80 °C, response is only 70 % of the response obtained at 55 °C, what follows the known relationship of partition coefficients vs. temperature.

**Fig. 6:**
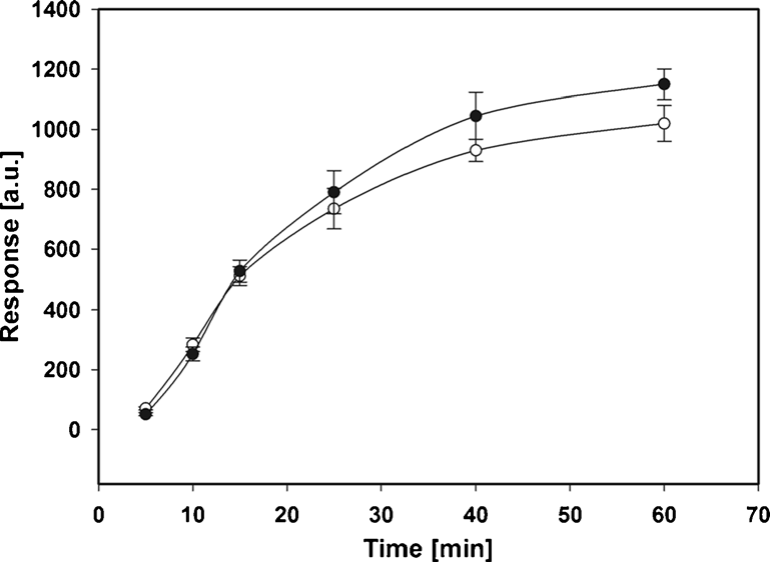
Dependence of the extraction efficiency (the sum of chromatographic peak areas) on the extraction time for the PCL/PDMS (*filled circle*) and DVB/PDMS (*open circle*) fibers (under optimized conditions)

At lower temperatures, PCL has the consistency of hard wax which may interfere with the diffusion of analytes into the coating. In the next analytical step, the extraction time profiles were determined for both fibers. The obtained results are presented in Fig. 6.

It can be noticed that the equilibrium state in both commercial and PCL/PDMS fibers was reached above 60 min of extraction. After about 25 min, the amount of retained analytes equaled ca. 75 % of that at 60 min. Therefore, the extraction time used in all subsequent experiments was set to 25 min. Also, PCL/PDMS fibers reveal higher efficiency than the DVB/PDMS ones at times above 15 min.

### Linearity, detection limits, and repeatability of the analytical procedures

For both fibers, the linear range was determined by conducting the extraction under optimum conditions (in triplicate) from samples at the concentration level ranging from 0.1 to 25 μg L^−1^. The detection limits were established based on the signal-to-noise ratio of 3:1. The measurement repeatability was calculated based on the measurements from five extractions in a sample at the concentration of 5 μg L^−1^. The obtained results are listed in Table 6. The calculated *R* ^2^ values were similar for both fibers and exceeded 0.98. This finding confirmed the existence of a linear relationship between the amount of retained analytes and their concentration in a sample for the investigated concentration range. The values of measurement repeatability for both fibers did not indicate any significant difference between them. However, the relative standard deviation calculated for the new fiber was slightly higher than that for the commercial fiber. The detection limits of the analytes determination with the PCL/PDMS fiber were slightly better than those obtained with the commercial fiber; both detection limits ranged between 0.12 and 0.75 μg L^−1^ (depending on the compound).

**Table 6:** Comparison of the values of linear range, detection limits, and extraction repeatability for the PCL/PDMS and commercial fibers

Compound	*R* ^2^		LOD (μg L^−1^)		LOQ (μg L^−1^)		RSD (%)	
	M-SPME	DVB/PDMS	M-SPME	DVB/PDMS	M-SPME	DVB/PDMS	M-SPME	DVB/PDMS
Ametryn	0.990	0.990	0.67	0.65	2.25	2.01	12	7
Atrazine	0.985	0.996	0.75	0.45	2.35	1.56	7	8
Prometon	0.997	0.988	0.15	0.75	0.48	2.66	11	8
Prometryn	0.986	0.990	0.50	0.31	1.85	0.94	13	12
Propazine	0.993	0.991	0.12	0.56	0.39	1.98	7	6
Simazine	0.992	0.995	0.52	0.44	1.60	1.23	8	8
Terbutryn	0.996	0.994	0.13	0.42	0.45	1.35	8	15

**Fig. 7:**
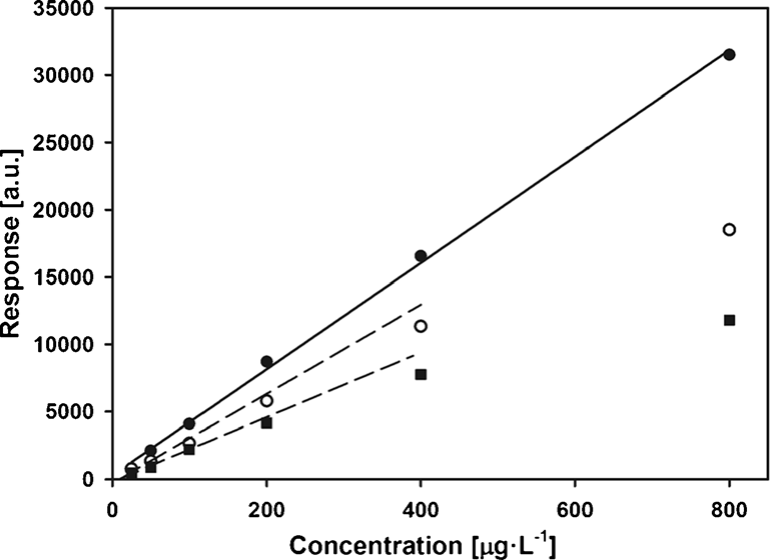
Calibration curves for triazines (sum of the peak area) for PCL (*filled circle*) and DVB/PDMS fibers. The latter ones determined in absence (*open circle*) and in the presence (*filled square*) of benzene. Benzene concentration equal to triazines concentration

Taking into account different mechanisms of extraction of analytes for M-SPME (adsorption) and commercial fiber (absorption), additional experiments were made. First, linearity ranges of calibration curves in analytes concentration level extending up to 800 μg L^−1^ were examined. In Fig. 7, dependences of sum of chromatographic peak areas vs. concentration of analytes in the sample obtained under optimal conditions are presented. As can be seen, there is a significant difference between both fibers. In case of the M-SPME fiber, calculated *R* ^2^ value for the investigated range is equal to 0.994 while for commercial fiber linearity of the relationships ends at analytes concentration equal ca. 200 μg L^−1^.

Another expected drawback of the utilization of the absorbents is their sensibility for matrix composition. This effect was studied by performing another set of extractions, where samples were modified by addition of benzene at the same concentrations as triazines. As it is shown in Fig. 6, presence of the benzene in case of the DVB/PDMS fiber significantly decreases the amount of the extracted analytes and narrows linearity range. Similar effect was not observed for PCL/PDMS (data not shown).

Although the main reason for using a PDMS membrane in the M-SPME fiber is to enable the application of liquids or substances miscible with water as sorbents, the membrane itself also takes part in the analyte isolation. The aim of the measurements was to determine the role of both sorptive phases in the M-SPME fiber in the process of analyte retention. For this purpose, the SPME fiber was coated with a polydimethylsiloxane with volume (0.40 ± 0.035 μL) similar to the volume of the membrane alone in the PCL/PDMS fiber (0.41 ± 0.04 μL). The extraction of analytes in samples at a concentration of 25 μg L^−1^ was conducted by using both fibers under conditions optimal for the PCL/PDMS fiber. The obtained results are listed in Table 7 as the areas of chromatographic peaks for all analytes.

**Table 7:** The peak area of analytes retained on the PCL/PDMS and PDMS fibers, and the percent share of the amount of analytes in specific phases

Compound	PCL/PDMS	PDMS	PCL	% PDMS
Ametryn	84	27	56	33
Atrazine	104	20	84	19
Prometon	105	28	77	27
Prometryn	86	30	55	35
Propazine	79	22	56	28
Simazine	143	20	123	14
Terbutryn	120	44	76	37

The peak areas of analytes retained in specific phases of the M-SPME fiber were calculated as differences between the peak areas obtained from the extractions using the PCL/PDMS and PDMS fibers. The percent share associated with retention of triazines on the membrane did not exceed 35 % despite the fact that the PCL volume present on the fiber had been only slightly (∼10 %) higher than the volume of PDMS. This finding confirms the high affinity of polycaprolactone for triazine compounds.

### Real sample analysis

In the final stage of research, the developed PCL/PDMS fiber was used to isolate triazines from real samples, i.e., samples of river and tap water. The results of analyte isolation from the sample of distilled water (pH 7 and 30 % NaCl) were used as a reference. The extractions were conducted for 25 min in samples brought to 70 °C. As no analytes had been detected in original samples of all three types of water, all samples were spiked with analytes to bring the concentration value to 5 μg L^−1^ (of each analyte). The obtained results are presented in Table 8. The results obtained indicate some matrix effects. Amount of analytes extracted from real samples are higher than those obtained for distilled water. The lowest influence of the matrix on the extraction efficiency is observed for prometryn while the highest for the trebutryn. On the other hand, all results are of the same order of magnitude that, on the very low concentration level, does not impair usability of the new fiber.

**Table 8:** Determination of triazines in real samples using the PCL/PDMS fiber (see text for details related to extraction)

Compound	Water sample		
	Distilled	Tap	River
Ametryn	18.4	31.3	28.5
Atrazine	15.8	18.5	21.5
Prometon	15.0	18.5	36.4
Prometryn	24.9	30.4	31.7
Propazine	20.0	19.8	31.2
Simazine	18.7	22.6	31.6
Terbutryn	9.7	29.7	32.3

## Conclusions

The results of the study on the application of polycaprolactone as a sorbent for isolating polar organic compounds from aqueous samples by means of the M-SPME technique are presented. Based on the determined McReynolds constants, which relate to molecular interactions characteristic for PCL, it has been established that PCL as a sorptive phase shows strong affinity towards triazine compounds.

The conducted screening analysis demonstrated that parameters such as sample temperature, salt content, and pH have statistically significant influence on the extraction efficiency. The optimum values of those parameters were determined for the extraction process with the application of the novel and commercial fibers. The optimization procedure was performed using the rotational central composite design. It was found that the influence of salt content and pH was similar in both fibers, i.e., 30 % NaCl content and pH 7. The influence of temperature on the isolation of analytes differed between the two fibers. The optimum temperature determined in this study was 60 and 70 °C for the commercial and PCL/PDMS fibers, respectively. Under optimum conditions, PCL/PDMS fiber revealed higher degree of linearity for the response variable as a function of analyte concentration in a sample. Comparison of the obtained detection levels of analytes demonstrated comparable or even slightly better properties of the PCL/PDMS fiber in relation to the commercial fiber, while the measurement repeatability was similar in both fibers. The applicability of the developed fiber has been confirmed on the basis of measurements in real samples, in which analytes were present at the concentrations recommended by the US EPA standards. Taking into account that triazines were used only for preliminary evaluation of the applicability of PCL as an extractant, the obtained results seem to be very promising. Presently, our team investigates possibility of using PCL/PDMS fiber for extraction of other classes of polar analytes from aqueous samples.
